# Challenges Associated with Smooth Muscle Tumor of Uncertain Malignant Potential (STUMP) Management—A Case Report with Comprehensive Literature Review

**DOI:** 10.3390/jcm13216443

**Published:** 2024-10-28

**Authors:** Jakub Kwiatkowski, Nicole Akpang, Lucja Zaborowska, Marcelina Grzelak, Iga Lukasiewicz, Artur Ludwin

**Affiliations:** 1 1st Department of Obstetrics and Gynecology, Medical University of Warsaw, 02-015 Warsaw, Poland; jakubkwiatkowski.uni@gmail.com (J.K.); nicoleakpang.uni@gmail.com (N.A.); zaborowska.lucja@doctoral.uj.edu.pl (L.Z.);; 2 Doctoral School of Medical and Health Sciences, Jagiellonian University Collegium Medicum, 31-530 Cracow, Poland; 3 Ludwin & Ludwin Gynecology, Private Medical Center, 31-511 Cracow, Poland; 4 Centermed Hospital and Clinic, 31-530 Cracow, Poland

**Keywords:** atypical leiomyoma, leiomyosarcoma, STUMP, smooth muscle tumors of uncertain malignant potential

## Abstract

**Background:** Smooth Muscle Tumor of Uncertain Malignant Potential (STUMP) is a poorly studied neoplasm that does not fulfill the definition of either leiomyoma or leiomyosarcoma. STUMP symptoms are indistinguishable from those of benign lesions; it has no specific biochemical markers or ultrasound presentations. The management of this type of tumor is particularly challenging due to significant heterogeneity in its behavior and the lack of clear guidelines; moreover, the lesion may recur after excision. **Case Report**: We report on a case of a 42-year-old patient diagnosed with a STUMP. The preliminary diagnosis was a submucous leiomyoma, which was removed hysteroscopically due to menorrhagia resulting in anemia. The histopathological examination of the resected myoma pointed to the diagnosis of STUMP. The hysterectomy was performed as the patient had completed her reproductive plans. There were no complications. The patient is currently recurrence-free after a 9-month follow-up. **Discussion and Conclusions**: The care of a patient diagnosed with STUMP requires a personalized approach and the cooperation of various medical disciplines, including molecular diagnostics, imaging techniques, and minimally invasive surgery. Management of STUMP must consider the patient’s plans for childbearing. All cases of tumors with “uncertain malignant potential” are a challenge in the context of patient-physician communication.

## 1. Introduction

Uterine smooth-muscle tumors are the most common gynecologic neoplasms, affecting more than three-fourths of women. Uterine leiomyomas are the most frequent benign tumors of the uterus, diagnosed in even up to 80% of perimenopausal women [1]. On the contrary, uterine sarcomas are considerably rare, occurring with a prevalence of 3–7% out of all uterine cancers in the American women population [2]. The spectrum of uterine neoplasms encompasses many borderline lesions that do not satisfy all the criteria for traditional diagnoses—one of them is STUMP, the smooth-muscle tumor of uncertain malignant potential. In the American women population, STUMPs comprise approximately 2–5% of all uterine smooth muscle neoplasms and occur in perimenopausal or postmenopausal women aged between 45 and 55 years [3]. STUMP’s five-year survival is estimated at 92–100% [4].

The definition of STUMP first appeared in 1973 and aimed to differentiate specific, poorly known malignant smooth-muscle tumors from well-recognized sarcomas [5]. The current World Health Organization (WHO) definition describes STUMP as a smooth muscle tumor with features that preclude an unequivocal diagnosis of leiomyosarcoma and do not fulfill the criteria for leiomyoma or its variants. These features raise concern that the neoplasm may behave in a malignant fashion [6]. STUMP cannot be characterized by specific clinical symptoms. Abnormal bleeding, anemia, fatigue, weakness, rapid tumor growth, and associated pain overlap signs and symptoms typical for leiomyoma, making the differentiation process even more challenging. Currently, there are no commonly recognized radiological or biochemical diagnostic markers or management guidelines. Since STUMP has a highly heterogeneous presentation and displays recurrence between 8.7% and 11%, each patient requires appropriate individual care. Studying this inadequately understood topic is essential for establishing transparent therapeutic strategies [7].

This study presents a case of a woman with histopathological STUMP in a submucous leiomyoma. The report discusses STUMP’s sonographic and clinicopathologic features and management following the current literature. The aim of the study is to critically analyze and summarize current knowledge about STUMP with emphasis on clinical presentation, utility of imaging methods, and pathological and immunohistochemical characteristics in the context of assessing malignant behavior risk. We also wish to review available treatment methods and follow-up schemes and compare literature-derived data with our case.

## 2. Case Report

A 42-year-old woman presented to the outpatient clinic of the 1st Department of Obstetrics and Gynecology of the Medical University of Warsaw. The main complaints were menorrhagia (menstrual bleeding for 10–14 days each cycle), dysmenorrhea, and mild anemia (Hb 10 g/dL). Heavy menstrual bleeding started about two years prior to the consultation and was initially treated with oral contraception combined with tranexamic acid and norethisterone acetate. One year later, the patient began experiencing excessive fatigue and was diagnosed with anemia. Ultimately, the patient presented to our outpatient clinic following an episode of severe bleeding that did not stop despite appropriate treatment. The patient had menarche at the age of 15 and was pregnant five times with two successful deliveries by cesarean section. She had no chronic diseases and had a normal Body Mass Index (BMI) (20.31). The patient denied a family history of cancer.

**Figure 1:**
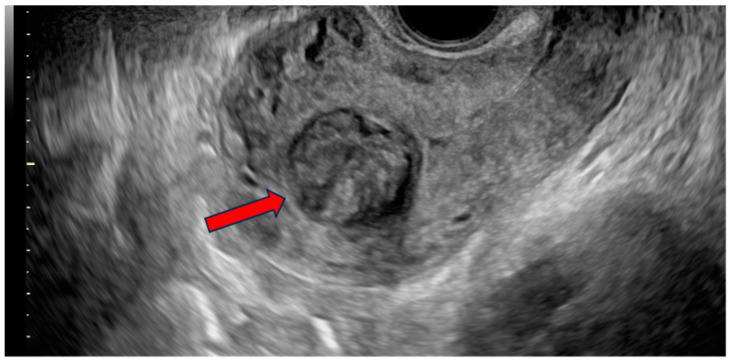
Transvaginal ultrasound (TVS) image showing a sagittal view of the patient’s uterus. A well-defined, solid lesion (red arrow) of mixed echogenicity is visualized within the borders of the uterine cavity. No evident acoustic shadow is present. The myometrium surrounding the lesion displays visible heteroechogenity with rich vascularization.

**Figure 2:**
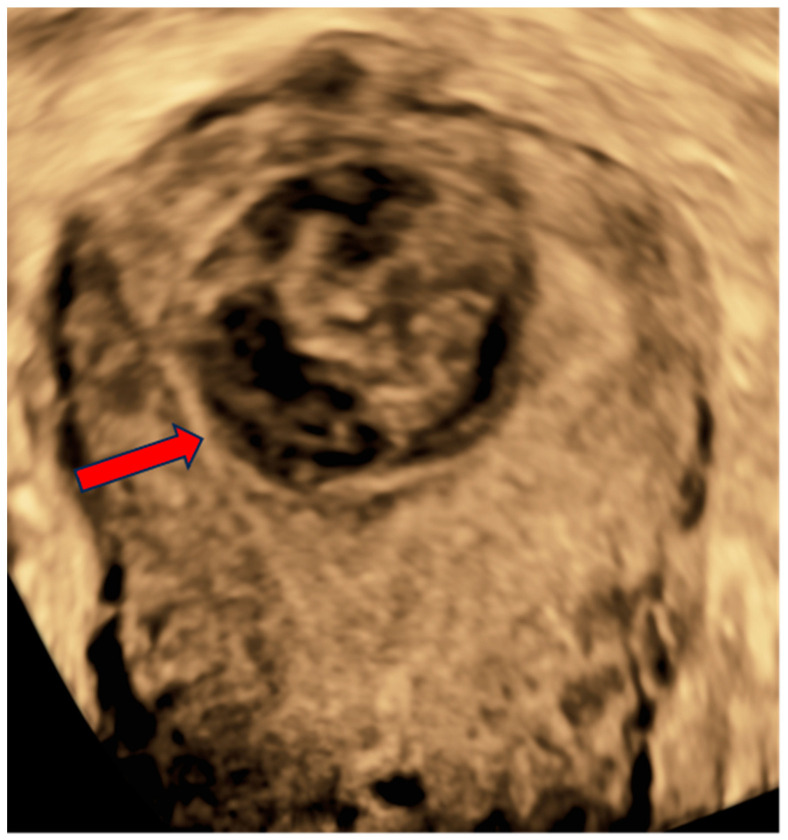
A coronal plane view of the patient’s uterus obtained with a 3D-TVS. The lesion (red arrow) protruding from the uterine fundus exerts a visible mass effect on the endometrial cavity.

On examination, there were not any palpable masses in the abdomen. The uterine corpus was not enlarged, not painful, and mobile. The cervix was normal-looking, although there was some bloody discharge and blood clots in the vagina. The adnexa were not palpable per vaginal examination. A two-dimensional/three-dimensional transvaginal ultrasound (2D/3D-TVS) examination using a Voluson E10 (GE Healthcare Ultrasound, Milwaukee, WI, USA) revealed a single oval-shaped submucosal lesion located within the uterine fundus, protruding into the uterine cavity (Figure 1 and Figure 2). The lesion measured 32.2 × 29.4 mm and had mixed echogenicity and well-defined borders without acoustic shadow. No calcifications or cystic areas were visible in the lesion. The uterus was anteverted, measuring 64 × 68.9 mm, with a thin endometrium and signs of adenomyosis within the myometrium. The adnexa was normal bilaterally, and there was no fluid in the Douglas pouch. The examination was performed by level three experts according to the European Federation of Societies for Ultrasound in Medicine and Biology (EFSUMB, www.efsumb.org, accessed on 20 October 2024).

A preliminary diagnosis of submucosal uterine leiomyoma was made, and a hysteroscopic removal of the lesion was planned. The lesion was successfully removed hysteroscopically in November 2023 without complications. The histopathological evaluation was performed by a pathology specialist, the head of the histopathology department, with 27 years of experience. The report described a mitotic count of 4 mitoses/10 high-power fields (HPFs), including atypical mitoses and moderate-to-severe diffuse cellular atypia. Coagulative tumor cell necrosis was absent. Based on the presence of cellular atypia and mitotic activity of <10 mitoses/10 HPFs, the final diagnosis was STUMP—Smooth Muscle Tumor of Uncertain Malignant Potential, spindle-cell type with high cellularity. The tumor was further categorized as high risk due to its immunoprofile—it was highly positive for p16 with a Ki-67 index of 30%.

In January 2024, the patient underwent Laparoscopy-Assisted Vaginal Hysterectomy (LAVH) with bilateral salpingectomy as she had completed her childbearing plans. The operation was performed without complications. The histopathological evaluation did not raise any concerns; no infiltration or vascular invasion features were found in the uterus and parametria. The uterine corpus showed features of internal adenomyosis and contained small intramuscular myomas of typical histology. No adjuvant therapy was undertaken. A follow-up schedule for this patient includes appointments at three-months intervals, during which a gynecological examination and transvaginal ultrasound are performed. Twelve months post-surgery, the patient is scheduled for vaginal vault cytology and contrast-enhanced computed tomography (CECT) of the thorax, abdomen and pelvis. After a follow-up period of 9 months, the patient remains disease-free.

The case summary is available in Table 1.

## 3. Discussion

Our case describes a STUMP diagnosis made after total hysteroscopic resection of a submucous leiomyoma performed in a woman with excessive uterine bleeding and accompanying anemia. Our patient’s age stands in accordance with the median age of STUMP diagnosis, estimated at 43 years [8]. The displayed clinical features of STUMP were concurrent with those of the typical presentation of a patient with leiomyoma. However, the differential diagnosis could include many more common pathologies, such as endometrial polyps, endometrial hyperplasia, endometrial carcinoma, abnormal uterine bleeding in perimenopausal women, and other types of smooth muscle tumors [10]. The main symptoms characterizing the disease are those related to abnormal uterine bleeding and pelvic mass—pelvic pain and symptoms of anemia, such as excessive fatigue and weakness reported by the patient [9].

There is no consensus on diagnostic criteria for STUMP. Clinically, STUMP is recognized when the Stanford criteria for leiomyosarcoma are not satisfied, but some features still indicate the malignant character of the lesion. The lesion has to display only one out of three available criteria: diffuse moderate-to-severe atypia, a mitotic count of at least 10 mitotic figures/10 HPFs, or tumor cell necrosis [13]. Tumors that meet two or more criteria are further classified as sarcomas. STUMP, like other uterine smooth muscle tumors, has several histological subtypes, of which the most common and best known is the spindle-cell type [16]. This particular subtype was diagnosed in our case. These tumors exhibit a wide range of presentations regarding expected size and location, with measurements varying from 0.7 cm to 18 cm [17]. These lesions can be situated within the cervix, the uterine corpus (intramural), or outside the uterine wall (subserosal). The most typical localization is intramural within the body of the uterus [3]. Our case is relatively rare due to its submucosal localization and a diameter of about 3 cm.

In the Stanford study, Bell divided problematic uterine smooth muscle cell tumors into four groups [18]:

- AL-LE—atypical leiomyoma (limited experience):

The tumor presents focal or multifocal moderate to severe atypia; there are less than 20 mitoses/10 HPFs and no coagulative tumor cell necrosis;

- AL-LRR—atypical leiomyoma, low risk of recurrence:

The tumor presents diffuse moderate to severe atypia; there are less than 10 mitoses/10 HPFs and no coagulative tumor cell necrosis. Our case can be assigned to this subgroup;

- SMT-LMP—smooth muscle tumor of low malignant potential:

The tumor shows no or mild cytological atypia, less than 10 mitoses/10 HPFs, and there is coagulative tumor cell necrosis;

- MAL-LE—mitotically active leiomyoma (limited experience):

The tumor shows no cytological atypia or coagulative tumor cell necrosis, but there are 20 or more mitoses/10 HPFs.

The heterogeneity of STUMP has raised the question of whether the frequency of tumor malignant behavior (meaning that it reappeared either locally or metastasized after a specific period) depends on the subgroup to which it can be assigned. In the study conducted by Ip [16], based on his team’s findings and previous research, examples of clinical malignancy of STUMP were found in 3 of the 4 subsets of STUMPs (AL-LE, AL-LRR, SMT-LMP). In this study, the risk of malignant behavior varied depending on the subgroups: 17.4% in AL-LE (4 of 23 cases), 26.7% in SMT-LMP (4 of 15 cases), and 4% in AL-LRR (2 of 50 cases). Due to the very limited number of cases, it cannot be determined whether the risk of recurrence significantly varies among these four subgroups—further scientific research is needed to clarify this. Dividing STUMP into subgroups may be helpful in pathomorphological diagnosis—but not in everyday clinical practice [16,17].

Another clinical issue concerns the assessment of tumor features and their possible link to the risk of malignancy in STUMP. The results of the available studies are inconclusive. According to Travaglino [18], in a study based on 219 STUMP cases, Stanford parameters used in STUMP diagnosis may assess the recurrence risk of STUMP. Moreover, significant cellular atypia and coagulative tumor cell necrosis, but not high mitotic index, can be stand-alone risk factors for the recurrence of STUMP [18]. In a systematic review from 2024 [3] that reported 99 cases of STUMP, no statistically significant associations were found between recurrence risk and high mitotic index, severe cytologic atypia, or presence of coagulative tumor cell necrosis. Despite reviewing the literature from the previous 20 years, the number of STUMP cases with documented recurrences was limited, which may have influenced the outcome of the statistical analysis. Based on available evidence, these features are probably not directly related to the risk of recurrence. However, many experts claim that the probability of malignant behavior can be successfully assessed based on the histological grade of STUMP [3,18,19]. Coagulative tumor cell necrosis seems to be the strongest predictor of malignant STUMP behavior and is often highlighted in studies as a notable indicator [16,17,19,20].

There are no ultrasound features that could specifically indicate malignancy in uterine smooth-muscle tumors [11]. Distinguishing STUMP from leiomyosarcoma based on imaging methods is virtually impossible. However, due to the widespread availability of ultrasound, these lesions are often visualized and described using this method. The most common morphological ultrasound features indicating the risk of malignancy are irregular borders of the lesion, irregular cystic areas, non-uniform echostructure, mixed echogenicity, and rich vascularization [21]. Malignant lesions are more commonly single, bigger than benign, and exhibit rapid growth.

On the contrary, visualization of normal myometrium and acoustic shadow is more typical for benign lesions [21]. The presence of calcifications is not indicative of the malignancy of the lesion; however, some authors suggest they are more common in benign leiomyomas [22]. In general, ultrasonography is not a perfect method to differentiate benign and malignant lesions with moderate specificity and sensitivity [23]. However, the use of these ultrasound features in combination with clinical data, such as age and presence of symptoms, in the form of diagnostic algorithms can be effective as a sensitive method of selecting patients at risk for STUMP or sarcoma with high negative predictive value, but at the cost of significantly lower specificity [21]. It is particularly challenging to differentiate sarcomas and STUMPs from degenerating leiomyomas with heterogeneous echogenicity and central necrosis. In our case, the ultrasonographic appearance was not characteristic of malignant lesions, although the lack of acoustic shadowing, mixed echogenicity of the lesion and rich vascularization are more frequently seen in STUMPs and leiomyosarcomas [21].

Magnetic Resonance Imaging (MRI) is used as a second choice in the radiological diagnosis of uterine tumors, with acceptable accuracy [24]. As necrosis or hemorrhagic content is often found in sarcomas or STUMPs, hyperintense signals on T1- and T2-weighted imaging MRI sequences can be associated with the potential malignancy of the lesion [25]. Diffusion-weighted MRI (DWI) and contrast-enhanced MRI (CE-MRI) are applicable in evaluating borderline cases and for accurate differentiation between benign leiomyomas and STUMPs. Within these indications, CE-MRI appears to outperform DWI [1]. However, similar to ultrasonography, some histological changes in benign lesions, such as various degeneration patterns or the presence of fatty tissue, may also show a signal typically often observed in sarcomas and STUMPs [25,26]. It should be emphasized that STUMP is a heterogeneous tumor group, and not every lesion of this type has necrosis, which makes radiological assessment even more challenging.

There are no practical guidelines concerning STUMP’s management, but surgical treatment appears to be an appropriate first-line therapy [8]. Further management depends on the initial procedure and the patient’s need to preserve fertility, as STUMP is typically diagnosed inadvertently after histopathological analysis following primary surgery [11]. If a myomectomy or supracervical hysterectomy has been performed earlier, the patient must be referred for a second surgery, including a total hysterectomy and staging [9]. In our case, a hysteroscopic myomectomy was an initial intervention that led to a definitive diagnosis. The procedure was subsequently followed by Laparoscopy-Assisted Vaginal Hysterectomy (LAVH) as our patient had completed her reproductive plans. If the patient wishes to preserve her fertility, myomectomy with subsequent hysterectomy can be recommended [12,15]. It is essential to note that the risk of recurrence is related to the out-of-bag morcellation rather than to the choice of surgical approach (open surgery/minimally invasive surgery) [11]. This is of particular risk as there is often no suspicion of possible tumor malignancy during the initial surgery. While an abdominal hysterectomy or myomectomy may lower the risk of spreading tumor cells in women with undiagnosed malignancy, it is linked to higher morbidity compared to minimally invasive methods [27]. The effectiveness of in-bag morcellation has not yet been sufficiently studied and confirmed [27].

Various molecular pathways that regulate cell proliferation involve oncogene products or tumor suppressor proteins. P53 and p16 are the most common tumor suppressor proteins and are highly relevant in STUMP evaluation [14]. P16 attaches explicitly to the cyclin-dependent kinase CDK-4, blocking the activity of the CDK4-cyclin D complex, thus functioning as a negative regulator of the cell cycle. However, contrary to what one might presume, this protein’s pathological overexpression is associated with carcinogenesis [28]. P53, often referred to as the “guardian of the genome”, plays a crucial role in preventing genome mutations. In response to stress, it maintains integrity by activating DNA repair mechanisms, arresting growth, or even initiating apoptosis. When mutations occur in the TP53 gene, p53 loses its ability to effectively manage the critical processes mentioned, resulting in uncontrolled cell proliferation and the development of cancer [29]. Similarly to p16, the overexpression of p53 is associated with carcinogenesis.

Immunoprofile can support the assessment of recurrence possibility. Low progesterone receptor (<83%), diffuse p16, p53 expression, and high proliferation index are associated with higher risk [11,14]. Immunohistochemical markers are an expert-recommended way to assess the risk of STUMP malignant behavior [7,16]. Consideration of the complete histological characteristics of the tumor, namely the presence of necrosis, mitotic index and degree of atypia, together with immunohistochemistry, seems to be the most plausible way of predicting tumor malignant behavior, especially in the context of studies indicating that these features are less reliable separately [3]. Immunoprofile markers can be particularly helpful in identifying patients requiring a closer follow-up or more aggressive surgery but should not serve as individual prognostic markers [11,14]. A strong correlation with disease-free survival was observed for p53 and p16, whereas no association was identified for the Ki67 index, which has prognostic significance in many neoplasms [14]. According to Travaglino’s systematic review, both p53 and p16 had moderate prognostic accuracy in STUMP, as recurrence rates exceeded 50% in cases with abnormal expression patterns, while they were below 10% in cases with normal patterns [14]. However, these markers can be misleading. Abnormal expressions of p53 and p16 can also be seen in typical leiomyomas with infarct-type necrosis and in leiomyoma subtypes. Hence, it is important to note that immunohistochemical findings must always be assessed in conjunction with histomorphological features [30]. As p53 and p16 stand as markers indicating higher recurrence potential, progesterone receptors (PR) are typically expressed in leiomyomas and STUMPs but not in leiomyosarcomas. High PR and low p53 expression can potentially exclude the diagnosis of leiomyosarcoma [31]. Our case was classified as a high-risk STUMP due to very high Ki-67 (30%) and intense positive staining for p16 (+++). In STUMPs, the Ki-67 labeling index typically varies between less than 5% and up to 25%. Higher Ki-67 levels are associated with more aggressive tumor behavior [3].

STUMP’s recurrence rate is 8.7% to 11% [7]. The lesions can recur as either STUMP or leiomyosarcoma [4]. Recurrent tumors require surgical intervention and adjuvant therapy. Possible options include pelvic radiotherapy, hormonal treatment with medroxyprogesterone or GnRH analogs and doxorubicin–cisplatin chemotherapy [7]. A typical time to recurrence ranges from 2 to 194 months, predominantly in the uterus, pelvis, lungs, retroperitoneum, liver, and bones [9]. Notably, the younger age of STUMP diagnosis correlates with a higher risk of recurrence during follow-up [7]. These findings highlight STUMP’s unpredictable behavior and the need for better prognostic tools and clear follow-up guidelines [9,32]. Due to the unpredictable progression of STUMP, some authors propose a regular follow-up after surgical treatment, scheduling appointments every six months for the first 5 years, followed by annual check-ups for the next 5 years [20]. Additionally, due to the high incidence of pulmonary metastases, annual radiological screenings are also advised [9]. Nevertheless, it should be emphasized that STUMPs are tumors with a good prognosis, and their therapy with careful follow-up is most often successful. In our case, we intend to provide a 10-year follow-up with a full gynecological examination and transvaginal ultrasound assessment performed every visit. Because of the increased risk of recurrence, the patient is currently being monitored every 3 months. These intervals can be extended or shortened according to the clinical situation, results of imagining evaluation and the time of follow-up. Due to the risk of extrapelvic recurrence, contrast-enhanced computed tomography (CECT) of the chest, abdomen and pelvis and vaginal vault cytology will be performed 12 months after surgery.

STUMP is a poorly understood tumor whose recognition is further complicated by cases that do not fit the typical picture of the condition. The literature reports cases of STUMP occurring outside the uterus, in the lower genital tract. Slatter described two cases of vaginal STUMP with aggressive behavior [33]. The lesions fulfilled the criteria for uterine STUMP since there are no specific guidelines for the diagnosis of a vaginal STUMP [33]. One patient died because of the disease, and the other was diagnosed with tumor recurrence. Neither of the tumors demonstrated p53 aberrant expression or p16 overexpression despite malignant behavior. These reports raise doubts about whether such markers should serve for risk assessment in vaginal STUMP. It should be noted that STUMPs can also occur in men in various locations, e.g., in the retroperitoneum [34].

An additional underestimated difficulty in managing STUMP is patient contact. Managing patients with tumors of uncertain malignant potential is an immense challenge, particularly in the realm of communication, as it involves addressing the trust difficulties that arise with making an “uncertain” diagnosis [35].

To summarize the knowledge about uterine STUMP, we propose recommendations on its management based on our comprehensive literature review:

- Risk assessment of malignant behavior—given the STUMP heterogeneity and unpredictable behavior, each patient should be individually assessed based on all accessible tumor characteristics, both histological and immunohistochemical markers [3]. Coagulative tumor cell necrosis, severe cellular atypia and high mitotic index may indicate a higher risk [18]. The most significant immunophenotype markers to assess the risk of recurrence are p53 and p16. High risk is associated with positive staining for p53 and p16 [14]. It should be noted that all aforementioned features have to be considered together to obtain a reliable evaluation [3,30];
- Consideration of the patient’s reproductive plans—women who have finished their reproductive plans should undergo a total hysterectomy [9]. In cases of women wishing to preserve fertility, myomectomy with postponement of hysterectomy should be considered [12]. The individual risks and available treatment options should always be discussed with the patient;
- Choice of a treatment method—we recommend minimally invasive methods as they are associated with lower morbidity compared to laparotomy [27]. Morcellation should preferably be performed in-bag, although further evidence is needed to conclude its effectiveness [11,27]. The risks and benefits of minimally invasive intervention and possible morcellation should be discussed with the patient preoperatively;
- Long and close follow-up—care for patients diagnosed with STUMP should be meticulous, not only during the treatment process but also during follow-up appointments after the treatment completion, considering the unpredictable locations of potential metastases [4,7,9]. A follow-up scheme should be planned based on the risk assessment. During the first 5 years, the patient should attend at least two visits annually that include a transvaginal ultrasound and a full gynecological examination. During the next 5 years, at least one examination should be performed annually [9]. In high-risk cases, the appointment frequency should be greater [14]. Furthermore, due to a potentially high risk of recurrence, a follow-up scheme should encompass more advanced imagining methods, such as a contrast-enhanced CT scan of the chest, abdomen, and pelvis, as well as a vaginal vault cytology [9]. However, it is important to emphasize that the post-treatment care plan should be individually tailored to the patient’s specific needs and risk factors. It is essential to establish standardized follow-up protocols to support clinical practice;
- Multidisciplinary approach—STUMP is an excellent example of a rare, complex medical condition that requires the cooperation of physicians from different specialties [9]. The team should consist of gynecologists, gynecological pathologists and oncologists. Cooperation and exchange of knowledge between specialists are crucial to making the entire treatment process successful.

Our study has several limitations. The case report is limited by its typical single-patient design, which restricts the generalizability of the findings. Further research involving larger cohorts or controlled studies is necessary to confirm these observations and explore their broader applicability. STUMP is a rare neoplasm whose definition has evolved over time. The cornerstone of diagnosis lies in histopathological evaluation, which is inherently subjective and heavily reliant on the experience and qualifications of the pathologist [3]. Researchers have highlighted concerns regarding the potential for overdiagnosis of STUMP, suggesting that diagnostic criteria may, in some instances, lead to an overestimation of the tumor’s malignant potential [3]. This introduces a risk of bias, particularly in the absence of standardized diagnostic thresholds.

## 4. Conclusions

The presented case exemplifies the challenges associated with STUMP management and the inherent issues linked to uterine smooth-muscle tumor evaluation. Treatment of STUMP requires a personalized, patient-focused approach extending from initial surgical intervention to carefully planned follow-up care. Every lesion resembling benign leiomyoma in terms of symptoms and ultrasound features should be carefully evaluated to avoid the possible dissemination of a malignant process. Clarifying management guidelines and advancing radiological and histopathological knowledge are essential for decreasing the chances of recurrence and increasing the survival time associated with STUMP.

## Figures and Tables

**Table 1:** Case summary with comments.

Category	Patient characteristics	Comments
Clinical characteristics	42-year-old; BMI 20.3.	The age was typical for STUMP diagnosis (median age: 43 years old [8]). Currently, no risk factors for STUMP have been identified [9].
Signs and symptoms	Menorrhagia, dysmenorrhea, mild anemia.	The patient’s symptoms were uncharacteristic, and many common pathologies should be considered in the differential diagnosis [10].
Obstetric history (Gravidity, Parity, Abortions)	G5P2A3; all (2) deliveries by cesarean sections.	The potential impact of the tumor bulging into the uterine cavity on the patient’s frequent miscarriages cannot be excluded.
Ultrasound features	Single oval submucosal fundal lesion; mixed echogenicity, well-defined borders without acoustic shadow.	Ultrasonographic appearance was not suggestive of malignant lesions.
Diagnosis	Preliminary diagnosis was submucosal leiomyoma—STUMP diagnosis was made based on histopathological results.	Currently, a definitive diagnosis of STUMP can only be made based on histopathological evaluation [11,12].
Histopathological Examination	Spindle-cell type tumor with high cellularity, diffuse moderate-to-severe atypia, mitotic index = 4/10 HPFs with atypical mitoses, no coagulative tumor cell necrosis.	The tumor met only one (marked atypia) of three Stanford criteria for leiomyosarcoma diagnosis and was classified as STUMP [13].
Immunoprofile	p16 (+++); p53 (−); Ki-67 30%; PR (+++)	Immunoprofile assessment can be valuable in the prognostic assessment of STUMP. Abnormal expressions of p53 and/or p16 indicates a recurrence risk exceeding 50% [14].
Treatment	Laparoscopy-Assisted Vaginal Hysterectomy (LAVH) with bilateral salpingectomy.	Conventional surgical STUMP treatment involves hysterectomy or myomectomy in limited cases of women wishing to preserve fertility [12,15].
Follow-up	After 9 months, the patient stays disease-free with no signs of recurrence	The follow-up for our patient will extend to 10 years, particularly considering that STUMP most commonly recurs more than 5 years after initial treatment [9].

## Data Availability

The original contributions presented in the study are included in the article; further inquiries can be directed to the corresponding author.
